# The Treatment of Cancer

**Published:** 1904-08

**Authors:** M. B. Hutchins

**Affiliations:** Of Staff of Presbyterian Hospital, Atlanta, Ga.


### THE TREATMENT OF CANCER.*

BY M. B. HUTCHINS, M.D.,
Of Staff of Presbyterian Hospital, Atlanta, Ga.

The physician or surgeon who undertakes the treatment of cancer has a task in no part of which he may expect much from *Vis Medicatrix Naturæ.* Cases of spontaneous recovery, as seen in so many maladies, are almost unknown. It is a fight to the end and against all kinds of odds. The first obstacle is the dilatoriness of the patient through ignorance, fear, or mal-influence, in seeking competent medical or surgical service. The most insurmountable obstacle is the pathology of the disease.

The essence of its pathology is erratic unlimited cellgrowth and metastasis. Other growths may be quite clearly outlined, the carcinoma or sarcoma may hold its least dangerous site in the part visible or palpable. Cells resembling normal cells, or recessive in character, take on abnormal multiplication and increase under some as yet unknown stimulus, invading tissues to which they are alien, sapping the vitality of the normal parts, taking a place which they are not vigorous enough to sustain. Then follow ulceration and necrosis while growth progresses, and, in the majority of cases, drifting of some of these cells through the lymph of blood-channels to other points. This metastasis, or separation

*Read before the Medical Association of Georgia, at Macon, April 20, 1904.

of cells to be lodged elsewhere, is often one of the earliest occurrences.

Epithelioma of the face above the line of the mouth, certain rodent ulcer forms and a few of the more severe types never or rarely undergo metastasis.

A sponge with its cavities and canals filled with shot would fairly illustrate the pathology of cancer, especially as regards its lack of a limiting wall, the diffuseness of extension. Some of these shot drifting into adjacent sponges would represent metastasis.

Any degree of success in the treatment of cancer can only be attained where the disease is yet confined to a limited or comprehensive area. There is but one rule, and this absolute, governing the treatment, and this is remove or destroy all diseased tissue. Any treatment which falls short of this requirement must fail, does often result in added malignance. Stimulating semi- or non-destructive applications are particularly harmful, incomplete excision often worse than useless. Instrumental inoculation of free tissues is often the result of careless operating. Following the rule that all diseased tissue must be destroyed or removed, the method of treatment becomes to some extent a matter of choice with the patient, in the case of superficial localized disease, or of the physician's preference. The choice should be left to the patient where there is a fair chance of success with one of several methods. In all cases the patient should be honestly and clearly advised and then given the privilege of deciding, thus removing much of the doctor's responsibility.

Where there is a reasonable certainty of our being able to include all diseased tissues and a chance of obtaining satisfactory healing, excision is the best of all methods. Where the diseased area is so hemmed in by bone, cartilage or important structures which can not be removed, some other plan may need be chosen. Excision and closure of the wound is practicable and to be favored in epithelioma of the elastic parts of the face, neck and body, provided there is no suspicion of metastasis. Small, presumably localized, epitheliomata which can not, through limitations or patient's objections, be excised can be cured by the thorough application of caustic potash, which has a special affinity for cells of the epithelial type. Pastes, as of arsenic, I have long since abandoned, the prolonged pain not being compensated for by the result obtained. Plasters, as of the perennial, multitudinous quacks, are damnable.

For non-metastatic cases of skin cancer, where excision is impracticable, the X-ray is the best of all treatments and is often all that remains for the hopeless cases. Preliminary curetting or local excision is desirable in most cases prior to X-ray treatment, save where the disease is markedly superficial or where, on the other hand, it is too extensive. I am convinced by my experience of the last three years that, taken as a whole, the total of cancer cases of all kinds receive more benefit in various ways from X-ray treatment than from all others combined. All the smaller, and many of the larger, localized cases are curable, at least temporarily, by this means; its application gives no pain, save from burns which at times must be produced; the pain of the disease is often relieved and the scar of healing is the slightest possible.

As regards the superficial, reasonably believed to be localized epitheliomata or rodent ulcers, the choice between caustic, cautery, X-ray and knife, or a combination of methods, may be largely governed by the will of the patient. Localized sarcomatous growth may yield with astonishing readiness to comparatively simple management, or it may become virulent in its malignance, even where treated with all possible energy. The result depends upon the type and the thoroughness of treatment. Anything short of the most vigorous and radical measures involves great risks.

As a rule sarcoma does not do well after caustics, but a single seminecrotic open lesion may be cured with an acid caustic, as nitric, or the acid-nitrate of mercury, or the thermocautery. Caustic potash is less adapted to this class of cancers. X-rays often act like magic; again the disease yields only after being most severely burned. Even in the latter case we may hasten metastasis.

I must say in this connection that my experience does not agree with that of writers who speak of the danger that X-ray treatment will hasten metastasis in the carcinomata. It certainly is not true of the epitheliomata.

Carcinomatous growths should always be excised en masse with diseased glands and intervening tissues, where their situation offers fair hope of cure. Knife transplantation must be avoided; the lines of excision must be as far as possible away from visibly diseased borders. Caustic or cautery should never be used on a cancerous growth of any extent with the expectation of a cure. Plasters of quacks, so used, convict the user of barbarous inhumanity. Where there is a redundant tissue, it should be removed, even if our chief reliance is to be placed in the X-ray. Radium acts somewhat similarly to the X-rays, is more convenient to apply in some regions, yet too expensive to be had in sufficient quantity for effective use.

There is no internal treatment for cancer save such as renders the patient better able to resist the disease through sustaining or improving the general health.

Probably a majority of cases of cancer which come to the legitimate specialist, or to the surgeon, are already beyond cure. In this class of cases our treatment must either be entirely palliative or solely with the object of giving the pitiable creatures a chance for a little longer life, a little less misery, a fractional percentage of hope for cure. Before the day of X-ray treatment and possibly of radium, these cases were more hopeless than now. An operation on a far-advanced case at the patient's request may render the surgeon accessory to suicide. The use of a cancer-quack's plaster would be criminal. Caustic and cautery would have the most limited use. X-rays, following where practicable preliminary removal of all visible disease, offer the best promise of comfort and possible cure.

Certain painful, sloughing or overgrown cancers, may be removed surgically for temporary relief. Smaller lesions may be treated with caustic or cautery for the same purpose, but with greater risk of acceleration of growth. After either, properly comes the X-ray. It is in the far advanced cases that all sorts of "cures" find their field. When finally convinced of the gravity of the condition, the patient begins to grasp at every branch of promise, often the greater the "promise" of the "curer," the less the cure. Ignorant cancer-doctors need not be discussed before intelligent physicians. The "cures" usually not only do no good, but they often add to the sufferings of the victim and hasten the end.

In legitimate practice, scarcely a year passes without the announcement by some honest, perhaps, but over enthusiastic doctor of a new cancer remedy, with cures published. Of such were the pyoktanins of some years ago, formalin, a new way to use arsenic, even calcium carbide (from which acetyline gas is made); electric treatment, etc. X-rays were heralded as a cure for all, but this superabundant faith has now crystallized into quite well defined knowledge of their certain value as a remedy and the unwarranted enthusiasm of its earlier days has subsided into a reasonable judgment. Radium, radio-active substances, even radium water, appear last on the tragic scene.

And still we have no specific for cancer. A few have more diagnostic ability, perhaps, and the treatment in competent hands is more thorough, but neither in diagnosis nor treatment are the conditions properly met by a large class of physicians.

Knowing the horrors of the disease, the shame in its name, the often loathsomeness, the insurance ban in the family history, every physician should view with suspicion and treat either with the greatest tenderness or the most vigorous and radical measures lesions or growths evidently malignant. Above all things, irritating applications and stimulating manipulations must be avoided. Treatment should be absolutely soothing and protective until eradication can be attempted or competent advice can be obtained. Excise, where practicable, with a reasonably wide margin of apparently healthy tissue, including suspicious glands and tissues intervening. Change knives of slightest suspicion of contamination, for fear of transplantation. In operating on open lesions, sterilize the surface, sew gauze over it if neceseary, or thoroughly cauterize before beginning. In using caustics or the cautery destroy more than the visible disease.

Do not trust X-rays alone, save in the smaller cases, the cases which patients refuse to have treated otherwise and where other methods can not succeed. In these cases the results will not disappoint as often as with other methods.

Recurrences are the rule in cancer, whether through diseased cells left after treatment or a development *de novo* in the cicatricially changed tissues. In this latter possibility I have strong belief.

Nodules appearing in the skin at a distance from a cancer, as of the breast, or its former site, are probably secondary lesions and often occur beyond the margins of the widest possible Halsted operation. But even with these recurrences, we shall often have prolonged the patient's life and added comfort to the remaining days by proper operation.

In those cases where even palliation can not be expected from severe measures, we must rely upon X-rays when available, cleanliness, supporting constitutional treatment and remedies for the relief of pain.

The suffering victim, doomed to drag out even a few more days or weeks of life and pain, with hope all gone, should be allowed to put his affairs in order and then be kept as profoundly under the influence of opiates or other anodynes as may be necessary for the alleviation of his misery. The time for fear of the morphine habit is passed; morphine or cocaine locally and any other means of relief should be afforded, urged *ad libitum.* The pain will shorten life and wrack the nervous system far more than drugs will injure the patient, and they alone provide the *lethe* to which he is entitled.—*Charlotte Medical Journal.*

